# Detecting the Minimum Limit on Wheat Stripe Rust in the Latent Period Using Proximal Remote Sensing Coupled with Duplex Real-Time PCR and Machine Learning

**DOI:** 10.3390/plants12152814

**Published:** 2023-07-29

**Authors:** Qi Liu, Tingting Sun, Xiaojie Wen, Minghao Zeng, Jing Chen

**Affiliations:** 1 Key Laboratory of the Pest Monitoring and Safety Control of Crops and Forests of the Xinjiang Uygur Autonomous Region, College of Agronomy, Xinjiang Agricultural University, Urumqi 830052, China; yep_ohh@163.com (T.S.);; 2 Key Laboratory of Prevention and Control of Invasive Alien Species in Agriculture & Forestry of the North-Western Desert Oasis, Ministry of Agriculture and Rural Affairs, Urumqi 830052, China

**Keywords:** wheat stripe rust, latent period, minimum detection limit, proximal remote sensing, duplex real-time PCR

## Abstract

Wheat stripe rust (WSR) is an airborne disease that causes severe damage to wheat. The rapid and early detection of WSR is essential for the prevention and control of this disease. The minimum detection limit (MDL) is one of the most important characteristics of quantitative methods that can be used to determine the scope and applicability of a measurement technique. Three wheat cultivars were inoculated with *Puccinia striiformis* f.sp. *tritici* (*Pst*), and a spectrometer was used to collect the canopy hyperspectral data, and the *Pst* content was obtained via a duplex real-time polymerase chain reaction (PCR) during the latent period, respectively. The disease index (DI) and molecular disease index (MDI) were calculated. The regression tree algorithm was used to determine the MDL of the *Pst* based on hyperspectral feature parameters. The logistic, IBK, and random committee algorithms were used to construct the classification model based on the MDL. The results showed that when the MDL was 0.7, IBK had the best recognition accuracy. The optimal model, which used the spectral feature R_2nd.dv ((the second derivative of the original hyperspectral value)) and the modeling ratio 2:1, had an accuracy of 91.67% on the testing set and 90.67% on the 10-fold cross-validation. Thus, during the latent period, the MDL of *Pst* was determined using hyperspectral technology as 0.7.

## 1. Introduction

Wheat is one of the most important crops in the world and has a pivotal position in ensuring food security [1]. Wheat stripe rust (WSR) is a typical airborne disease that seriously affects wheat yield and quality [2,3,4]. WSR is caused by *Puccinia striiformis* f.sp. *tritici* (*Pst*), a living obligate parasite [5]. When wheat is infected by *Pst*, the parasite enters a latent period, during which the wheat is generally not symptomatic. However, during this period, a parasitic relationship is established between the host and *Pst*, which can absorb water and nutrients from the host. Although there are no symptoms during this period, the infection results in a series of metabolic changes at the host’s diseased site, causing changes in the water and pigment content of the host cells [6]. In the late latent period, the wheat leaves will form spots and then produce bright yellow spore piles, which are arranged in narrow stripes that run parallel to the leaf veins [7]. When the first spore breaks, it means that the latent period of the WSR is over, and the wheat has entered the symptomatic period of WSR [8]. During WSR epidemics, the latent period has significant research value in terms of the infection process because it poses a strong risk of uncontrolled spread. If *Pst* can be detected during the latent period, it would be beneficial in reducing disease loss and improving crop quality.

The traditional estimation of disease severity Is conducted by field scouting where diseased wheat is already showing obvious symptoms, and it is difficult to control WSR. Field scouting is time-consuming, laborious, and has low accuracy [9]. The naked eye cannot identify the latent period of WSR because it does not show obvious symptoms. Therefore, a method that can early, quickly, accurately, and quantitatively identify WSR during the latent period is needed [6].

At present, nucleic acid detection is the most commonly used method to estimate the content of pathogens in the laboratory, such as the polymerase chain reaction (PCR) [10,11], loop-mediated isothermal amplification method (LAMP) [12,13], and CRISPR/Cas-based detection method [14,15], which have risen rapidly in recent years. These technologies have been demonstrated to have reliable and high detection accuracy but generally depend on precision instruments and expensive reagents. This not only requires lots of labor costs due to complicated pre-processing but also requires a skilled operator [16]. In addition, nucleic acid detection methods have high requirements for the detection environment, sampling, nucleic acid extraction, and obtaining detective results; each step is faced with the risk of pollution, especially in the field [17]. Meanwhile, these methods are a form of single-point detection of plant pathogens, which cannot be used for large-scale and high-throughput detection in the field [16].

With the continuous advancement of information technology, proximal remote sensing (PRS), an emerging technology, has been used more and more widely in agriculture. Varied plants have different spectral properties because of their unique morphology and composition, which makes them noticeably different from the spectral information of other ground objects [18,19,20,21,22]. The extensive spectrum data produced by plants’ ongoing interaction with environmental elements, such as biological and abiotic factors, during the growing process is what makes up their typical spectral features, such as starches [23,24,25], cytochromes (chlorophyll [26,27]), water [28,29], and carbohydrates [30,31]. The contents of these components will vary between plants or even within the same plant under various growing conditions, and these variations will be shown in the plant’s reflection spectrum. Therefore, by detecting the spectrum information of biochemical substances, PRS may retrieve the content of diverse biochemical components [32].

Plant diseases will alter a plant’s exterior morphology and internal physiological composition, which will modify the plant’s physical or chemical characteristics and, in consequence, alter its spectral properties. For instance, the disease index of WSR was associated with the canopy reflectance in the 630–687 nm, 740–890 nm, and 976–1350 nm bands that were thought to be the sensitive bands [33]. In the band of 376–1600 nm, the spectral reflectance of individual *Pst*-infected wheat leaves was positively linked with the severity of the disease. The correlation peaked in the bands of 446–725 nm and 1380–1600 nm [34]. Therefore, by detecting variations in the spectrum, PRS will be able to pinpoint the related stress factors [35,36,37,38,39,40]. And it is possible to quickly and non-destructively obtain the hyperspectral characteristics of objects in a large space.

At present, PRS is widely used to detect and identify whether or not wheat is diseased [41,42]. Qualitative monitoring is also a hotspot in hyperspectral research, but a few studies have performed quantitative analysis based on the severity of WSR with PRS [43,44,45]. The quantitative detection of WSR in the preceding article, however, was based on the symptomatic period rather than the latent period. Yet, if the severity of the disease was detected early enough in the latent period, this would reduce the cost and improve the efficiency of controlling the disease. Alternatively, quantitative detection is conducted in accordance with the aberrant alterations in the color and texture of wheat leaves after *Pst* infection rather than focusing on the quantity of pathogens. Therefore, in order to fill the gap in the literature, an effective, economical, and precise quantitative method for WSR detection during the latent period should be developed.

The novelty of this work mainly includes three points as follows: 1. It established a rapid detection model for WSR by combining PRS and a quantitative PCR. 2. It found that the minimum MDI corresponding to wheat stripe rust at the latent period can be detected via PRS, namely, the minimum detection limit (MDL). 3. The two results above were helpful to improve the quality of hyperspectral remote sensing monitoring from qualitative to quantitative analysis and also lay a foundation for further research on the correlation of spectral characteristics to plant physiological composition changes caused by stripe rust. At the same time, the MDL also directly defines the range of quantity for predicting the occurrence degree and harm severity of WSR in field wheat.

In previous research, the method to detect WSR during the latent period using a machine learning model based on hyperspectral data had been established [46]. In this study, PRS was used to realize the quantitative detection of WSR in the latent period. The objectives to reach this goal were (1) detection of the MDL based on the canopy spectral reflectance data and (2) constructing and assessing the detection model of WSR in the latent period based on the MDL values with machine learning.

## 2. Results

### 2.1. Correlation Analysis of MDI and DI

**Figure 1:**
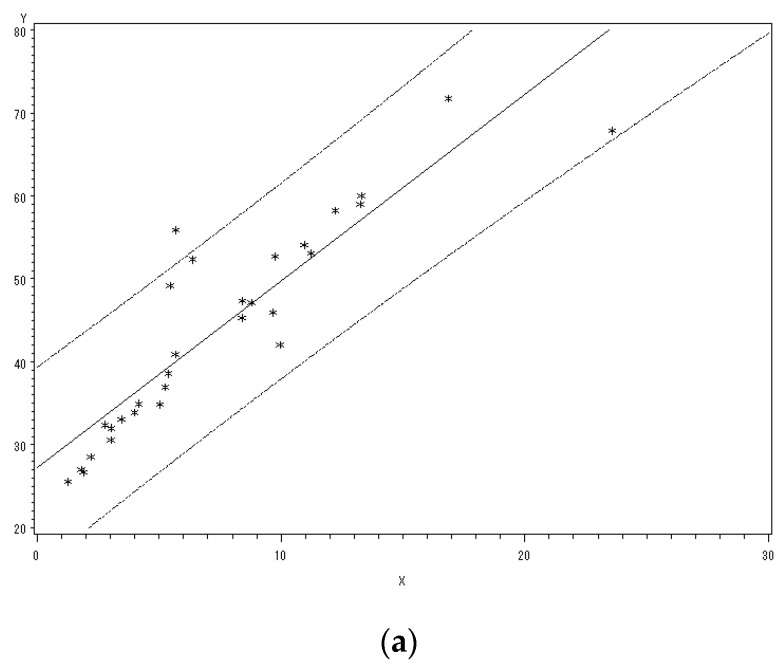

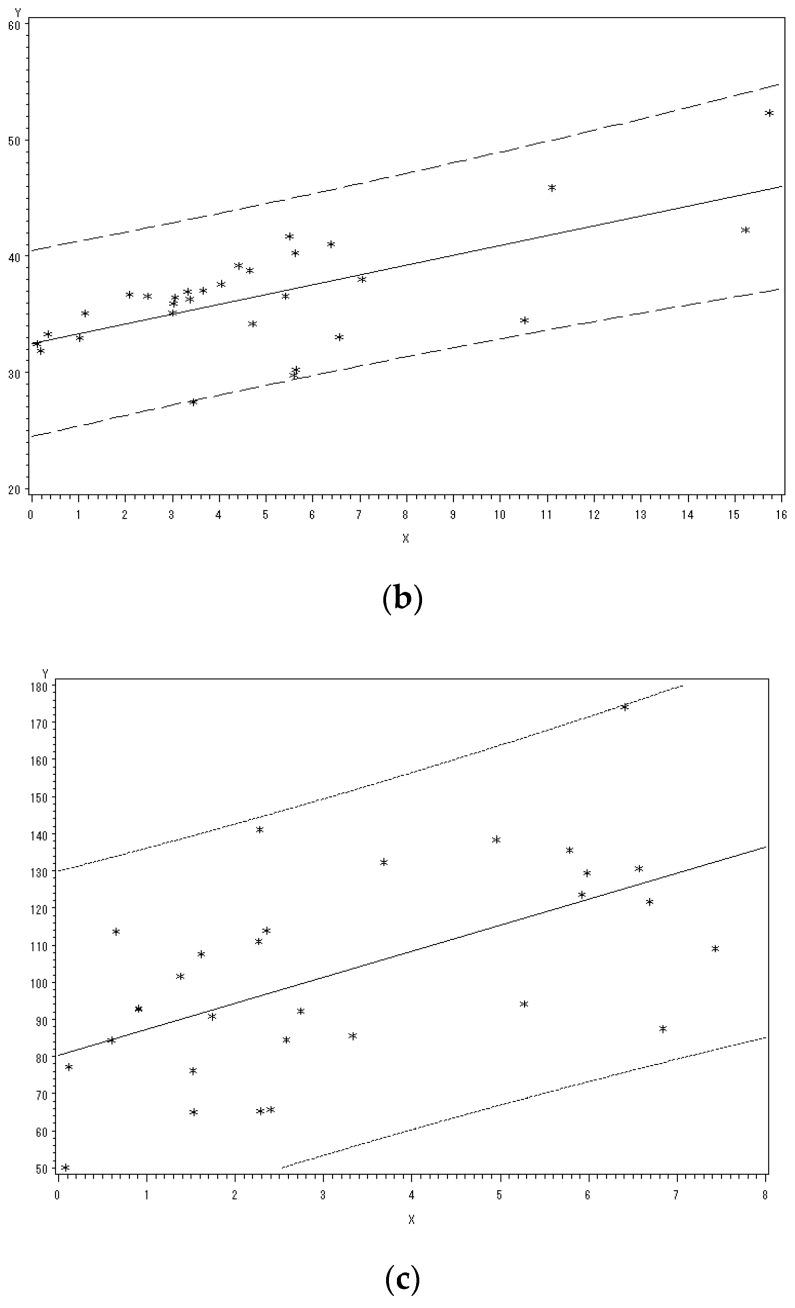
(**a**) Correlation between MDI-AUDPC and DI-AUDPC in Mingxian169. (**b**) Correlation between MDI-AUDPC and DI-AUDPC in Jing 0045. (**c**) Correlation between MDI-AUDPC and DI-AUDPC in Nongda 195.

A correlation analysis between the MDI-AUDPC and the DI-AUDPC of the three varieties of Mingxian 169, Jing 0045, and Nongda 195 was performed. The results are shown in Table 1 and Figure 1.

The correlation between the MDI-AUDPC and DI-AUDPC of the three wheat cultivars was very significant; the higher the *Pst* susceptibility of wheat varieties, the higher the correlation coefficient. Therefore, the MDI during the latent period could be used to predict the DI in the symptomatic period.

### 2.2. Hyperspectral Curves of Different Varieties at Four Sampling Times

**Figure 2:**
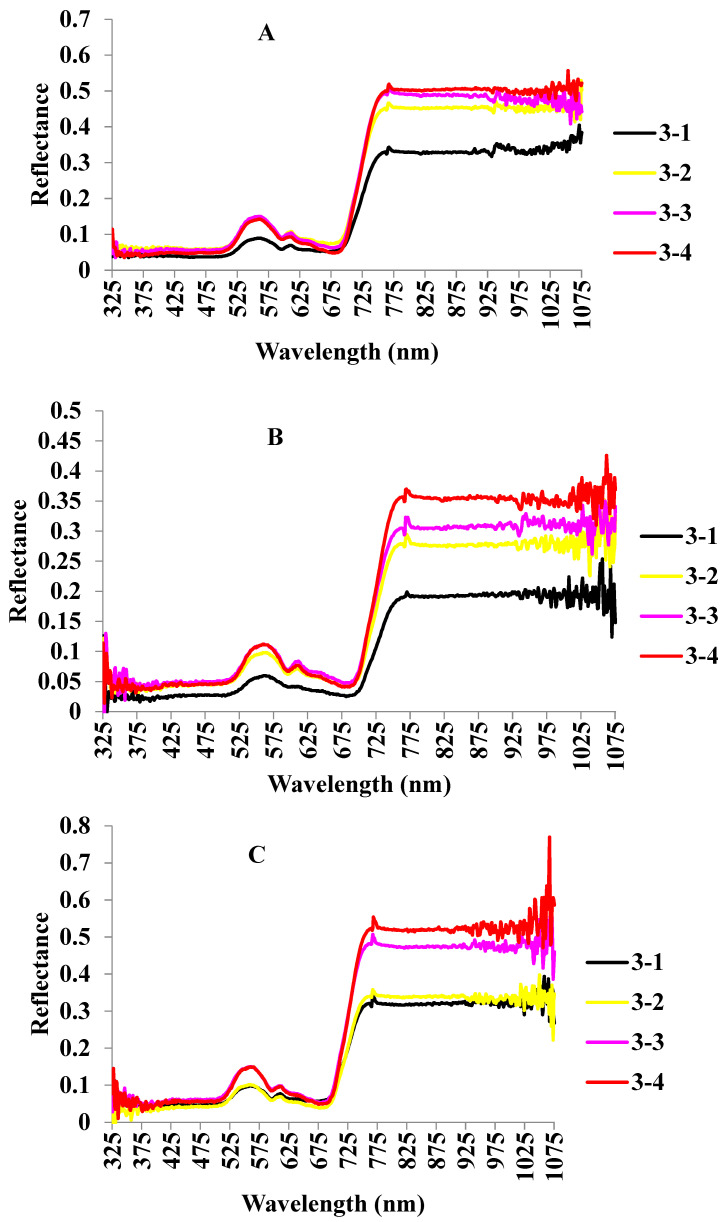
Hyperspectral curve of different wheat varieties at different times. Note: (**A**) Mingxian169, (**B**) Jing 0045, (**C**) Nongda195, 3-1 indicates the first day before inoculation, 3-2 indicates the third day after inoculation, 3-3 indicates the fifth day after inoculation, and 3-4 indicates the eighth day after inoculation.

The development of the canopy spectral profiles of different cultivars at four sampling times was observed. Each spectrum was obtained from the averaged spectra of 30 samples under the same inoculation concentration on the same day. Three wheat varieties with different levels of resistance had similar canopy spectrum variations. The development of the spectral profile is shown in Figure 2. The fastest changes are noted for Mingxian169 (highly susceptible to *Pst*), while the slowest for Nongda195 (highly resistant to *Pst*). The decrease at the red valley (near 680 nm) and the increase at the green peak (near 550 nm) were noticeable. The band of chlorophyll absorption was 680 nm and indicated that the chlorophyll content was declining. The 700–780 nm band was referred to as the “red edge region” of vegetation reflectance. With the increase of the latent time, the reflectance increased rapidly, indicating that the wheat was affected. The 780–1050 nm band mainly reflected the internal structural characteristics of the leaves. Its reflectivity increased with the increase of the latent time and reached the highest level on the eighth day, which showed that the interior structures of the wheat cultivars with different levels of resistance all changed during *Pst* infection. Figure 2 further illustrates that PRS is feasible for the detection of the latent period of WSR.

### 2.3. Detection of the MDL Based on the CART Algorithm

The analysis of the MDL based on 24 testing sets is shown in Table 2. When the modeling ratio was 1:1 based on R and lg(1/R), the MDL was much larger than the other 3 modeling ratios. Considering that there could have been classification errors, the average values of the MDL under the other three ratios were calculated. The MDLs were 0.7083 and 0.7093 based on R and lg(1/R), respectively. Based on R_1st.dv and R_2nd.dv, the decision tree failed to find the intersection point of the MDI when the modeling ratio was 1:1, while the average values of the MDL under the other 3 ratios were 1.214 and 1.2282 for R_1st.dv and R_2nd.dv, respectively. Based on lg(1/R)_1st.dv and lg(1/R)_2nd.dv, the average values of the MDL obtained under the 4 modeling ratios were 1.6903 and 1.2318, respectively. Based on the six transformed hyperspectral features, MDL analysis was carried out on the six complete datasets. The results are shown in Table 3. The data details could be checked in the supplementary materials (Figures S1–S7).

The MDLs of the six hyperspectral features and the average of the different modeling proportions were rounded off from the percentile shown in Table 4. The obtained MDL values could be divided into 4 categories: 0.7 (0.6939/0.7083/0.6939/0.7092), 1.2 (1.1718/1.2140/1.2193/1.2282/1.1560/1.2318), 1.7 (1.6903), and 1.3 (1.2984). Because 1.7 and 1.3 were not reduplicative, they were not used for further analysis. Therefore, the MDL values were 0.7 and 1.2, which are the amount of *Pst* that can be detected by PRS.

### 2.4. Classification Results of Different Models with Different Hyperspectral Features in the 325–1075 nm Waveband

**Figure 3:**
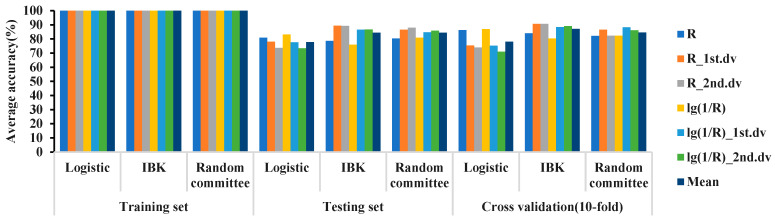
Classification accuracy for MDL of 0.7 in the 325–1075 nm waveband.

In the 325–1075 nm band, combined with the 6 hyperspectral features and 4 modeling ratios, the logistic, IBK, and random committee methods were used to establish classification models for the 2 detection limits of 0.7 and 1.2. The classification results for the MDL of 0.7 are shown in Figure 3. In the 325–1075 nm waveband, the accuracy of the training sets of all 3 models was 100%. The classification accuracy of IBK was better than that of random committee and logistic. The accuracy of the three models on the testing set was between 73% and 90%, and the accuracy of the 10-fold cross-validation was between 70% and 90%.

In the cross-validation results, the optimal model based on the 3 algorithms in the 325–1075 nm waveband is shown in Table 5. The accuracy of the training set of the optimal model was 100%, the accuracy of the testing set of the 3 models was between 85–91%, and the 10-fold cross-validation accuracy was 86–90%. The optimal model used R_2nd.dv, lg(1/R)_1st.dv, and lg(1/R) as the spectral features and 2:1 and 3:1 as the modeling ratios.

**Figure 4:**
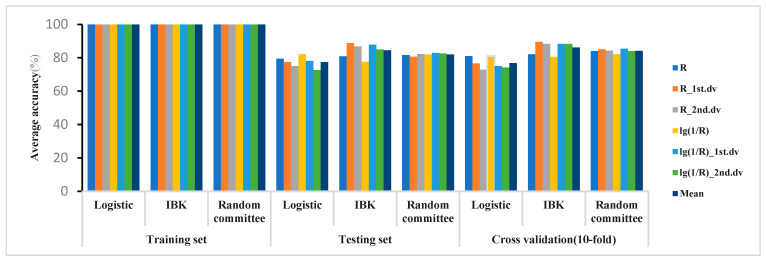
Classification accuracy for MDL of 1.2 in 325–1075 nm waveband.

The classification results for the MDL of 1.2 are shown in Figure 4. The results showed that in the 325–1075 nm waveband, the accuracies of the training sets of the 3 models were all 100%, and the accuracy of IBK outperformed random committee and logistic. The accuracy of the 3 models on the testing set was between 72% and 89%, and the accuracy of the 10-fold cross-validation was between 72% and 90%.

In the cross-validation results, the optimal model based on the 3 algorithms in the 325–1075 waveband was shown in Table 6. The accuracies of the training set were all 100%, the accuracy of the testing set was between 85% and 91%, and the 10-fold cross-validation accuracy was between 80% and 89%. The optimal model used R_1st.dv, lg(1/R)_1st.dv, and R as the spectral features and 2:1 and 1:1 as the modeling ratios.

**Figure 5:**
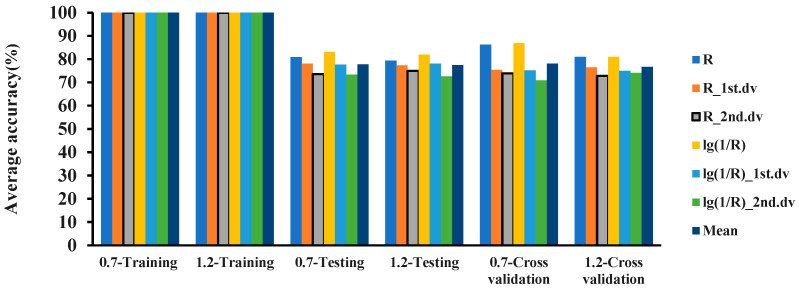
Classification accuracy of two detection limits based on the logistic algorithm in the 325–1075 nm waveband.

**Figure 6:**
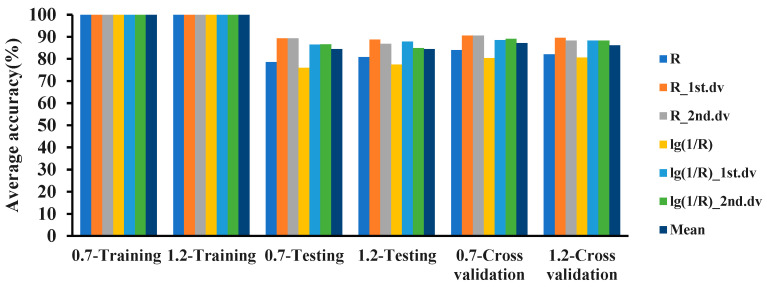
Classification accuracy of two detection limits based on the IBK algorithm in the 325–1075 nm waveband.

**Figure 7:**
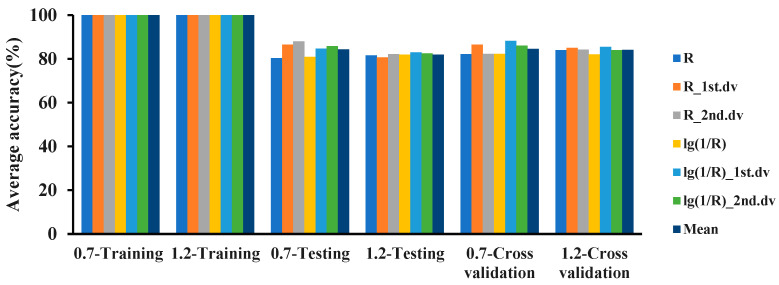
Classification accuracy of two detection limits based on the random committee algorithm in the 325–1075 nm waveband.

The classification accuracies of the 3 models built with the MDL values of 0.7 and 1.2 were compared. The results are shown in Figure 5, Figure 6 and Figure 7. In the 325–1075 nm waveband, the accuracy of the classification model with 0.7 as the MDL using the logistic, IBK, and random committee algorithms was different from the accuracy of the model with 1.2 as the MDL. The accuracies of the training sets of the 3 algorithms were all 100%. In the testing set, except for IBK, the average accuracy of model recognition with a 0.7 detection limit in the other 2 algorithms was higher than that of the model with a 1.2 detection limit. For the average recognition accuracy in the 10-fold cross-validation, the model recognition accuracy with a 0.7 detection limit was higher than that with a 1.2 detection limit. For the analysis of 6 spectral features, in the testing set and in cross-validation, the recognition accuracy of the model with an MDL of 0.7 was higher than the accuracy with an MDL of 1.2. Based on the above results, it could be concluded that the recognition accuracy of the models established with a 0.7 detection limit was better than that of the models established with a 1.2 detection limit. Therefore, the minimum molecular disease index that can be detected by PRS is 0.7, and the classification accuracy of IBK is the best.

## 3. Discussion

### 3.1. The Severity Assessment of WSR

WSR-infected wheat would exhibit a series of external morphological changes that occur in the symptomatic period and internal physiological changes that occur in the latent period. All of them would lead to spectrum changes. Through quantitative analysis of spectral variations of the foliar, PRS will have a doorway through which to locate diseased plants [47]. Recently, the majority of studies mostly on the severity assessment of WSR were conducted during the symptomatic stage. Guo et al. [42] used a combination of the spectral features and textural features of hyperspectral images to assess the damage levels in wheat leaves at the leaf scale, and the identification accuracy was up to 95.8%. Zhao et al. [43] used the ASD Leaf Clip to collect in situ hyperspectral data of wheat leaves showing symptoms of WSR and assess the severity of individual wheat leaves through water and chlorophyll content changes. Wang et al. [44] collected the hyperspectral data of wheat leaves in the latent period and symptomatic period of WSR under in-field conditions using a black paper as the background, and the disease severity was accurately retrieved using inversion models with an R^2^ of more than 0.90 and an RMSE of less than 0.15. Ren et al. [45] created a new spectral index (YROI) to quantitatively estimate the severity of WSR based on the spectral response of spores at the leaf scale (R^2^ = 0.822, RMSE = 0.070) and at canopy scales (R^2^ = 0.542, RMSE = 0.085). The methods used in the aforementioned articles to assess the disease severity were either based on the leaf scale, had poor performance at the canopy scale, or required external assistance to attain high assessment accuracy, which had certain limitations when applied to large-scale disease monitoring in the field.

In the meantime, in addition to Wang’s work, the prior publications assessed the severity of WSR at the symptomatic stage. However, the yellow rust spores are airborne and can quickly spread an epidemic in the surrounding wheat when the disease’s evident symptoms show up on leaves. Farmers will thereafter employ chemical agents in high doses and across a wide area, endangering both the environment and human health. So, it is still essential to monitor and forecast WSR as early as possible in order to prevent and manage WSR.

Although WSR has no evident symptoms in the latent period, in particular, changes in the water and pigment of the foliar are the most critical characteristics associated with the severity of the disease because water and pigments tend to robust plants [6]. Because the MDI is also used to evaluate the disease severity during the latent period, it is important and helpful in assessing the severity of WSR at an early stage. In our previous studies, we established a machine learning model using hyperspectral data to identify the WSR in the field during its latent period [46]. As a follow-up to this study, we want to find out whether PRS is capable of detecting the minimum MDI value. As a consequence, this study suggested the CART algorithm to detect the MDL and used machine learning and canopy hyperspectral data to identify the DI during the symptomatic period.

Although studies on disease early detection have been conducted for a long time, particularly those based on hyperspectral technology, these techniques have not yet been widely used in actual production [48]. It is possible to divide determining the disease severity using hyperspectral data into two categories: (1) choosing the most appropriate spectral indices based on external morphological changes and physiological (pigment and water content) changes and (2) using model fitting and classification to inverse the disease severity. In terms of determining the severity of plant diseases, each method offers benefits and drawbacks. The various techniques that can be combined to achieve complementary advantages in WSR latent period detection at the field scale are worth trying. This will continue to be the focus of our research going forward.

This study has only been conducted at the wheat canopy scale; thus, the next step should be to check the MDL’s accuracy at the field scale. Second, a precise inversion model is employed that combines hyperspectral data with the MDI of WSR in order to precisely assess the severity of WSR at the latent period. Finally, because the monitoring model for this study was developed in the 325–1075 nm band range and comprised an amount of data with redundant and invalid bands, more research is still needed to figure out the optimal waveband.

### 3.2. Classification Results Based on the two MDL

This research achieved good classification performance to identify WSR during the symptomatic period, and the accuracy of the model based on the 2 MDLs was 91.67 and 91.03, respectively. The intensity of the illumination and interference from the soil background may be the reason why there is still considerable space for improvement. To increase the model’s classification accuracy, further optimization of the model’s parameters, wavebands, and spectral characteristics should be carried out. The disease identification accuracy of this study was less accurate than that of Wang’s method [44], but a new technique for quantitatively monitoring disease severity has been devised, providing a foundation for widespread implementation in the field.

Rapid disease prevalence and the dissemination of plant pathogens are facilitated by human activities and global climate change [16]. As a result, it is crucial for early, large-scale, and accurate monitoring of disease incidence and severity. At the same time, the prediction model is integrated with local meteorological data and historical disease epidemic data, which is useful to increase the prediction accuracy of the spatio-temporal epidemic dynamics of WSR.

## 4. Materials and Methods

### 4.1. Experimental Material

The wheat cultivars used were Nongda 195 (highly resistant to *Pst*), Beijing0045 (moderately susceptible), and Mingxian169 (highly susceptible). The test strains used were three races of *Pst*, CYR32, CYR33, and V26, which were mixed in equal proportions. The concentration of the spore suspension used was 0.05 mg/mL. The Plant Disease Epidemiology Laboratory of China Agricultural University provided the above materials.

About 300 wheat seeds were planted in 1 flowerpot, which had a 33 cm × 26 cm area. Sterilized soil and humus substrate were placed in 1 flowerpot in a ratio of 1:1. There were five pots of each wheat variety inoculated with *Pst*, and one pot for each variety was used as a healthy control. Each flowerpot was divided into six sampling points. Each sampling point had an area of about 11 cm × 13 cm, which contained 45 wheat plants. After 15 days, the wheat was inoculated with the suspension, which was mixed with 3 mg *Pst* with 10 mL 0.05% tween solution. A clean finger was used to remove the wax from the leaves, and then a hand-held sprayer was used to evenly spray the urediospore suspension on the leaves. Finally, the inoculated wheat plants were placed in a foam box for dark treatment. After 24 h, the wheat plants were taken out and placed in a climate-controlled room.

### 4.2. Hyperspectral Remote Sensing Data Acquisition and Preprocessing

**Figure 8:**
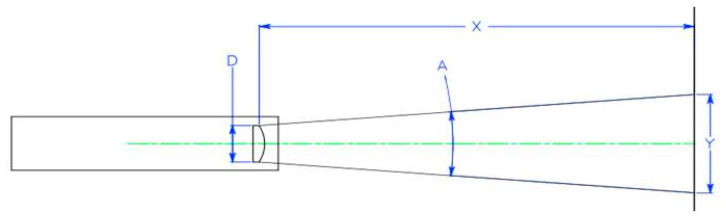
The ground sample distance schematic of ASD FieldSpec^®^ HandHeld™2 spectrometer.

The ASD spectrometer (ASD FieldSpec^®^ HandHeld™2, ASD Inc., Boulder, CO, USA) was used to collect the canopy hyperspectral data. The field-of-view of the spectrometer was 25°, the resolution was less than 3 nm, and the minimum integration time was 8.5 ms. The sample distance schematic of ASD spectrometer was shown in Figure 8. The spectrum average was set at 15. Three spectra were measured for each sampling point, and the average value was treated as the spectrum of the sampling point at the canopy level. Calibration with a white board was performed every 10 min to prevent the change of the sun’s incident angle and the systematic noise stemming from the instrument. All hyperspectral measurements were collected in cloudless and windless weather between 10:00–14:00 (Beijing time).

The area of the collection was 11 cm × 13 cm, containing 45 wheat plants, and the sampling vertical distance from the wheat canopy was 25 cm.

In this study, the latent periods of Beijing 0045 and Mingxian 169 were both 8 days. Wheat canopy hyperspectral data of these 2 cultivars were collected 4 times: 1 day before inoculation and on the 3rd, 5th, and 8th day after inoculation. The latent period of Nongda 195 was 10 days, and the wheat canopy spectrum was collected 5 times, including 1 d before inoculation and on the 3rd, 5th, 8th, and 10th days after inoculation. A total of 1404 canopy spectra of wheat were obtained, which included 324 spectra of healthy wheat before inoculation, 180 canopy spectra in the control, and 900 canopy spectra of wheat in the latent period of WSR. The 468 spectra were used for constructing the model.

In this study, the original hyperspectral data were processed through the ViewSpecPro software (ASD FieldSpec^®^ HandHeld™2, ASD Inc., Boulder, CO, USA), and six types of data transformation were used for hyperspectral data, including the original hyperspectral reflectance values (R), the first derivative of the original hyperspectral value (R_1st.dv), the second derivative of the original hyperspectral value (R_2nd.dv), the logarithm of the reciprocal of the original hyperspectral reflectance values [lg(1/R)], which is called the pseudo absorption coefficient (PAC), the first derivative of PAC [lg(1/R)_1st.dv], and the second derivative of PAC [lg(1/R)_2nd.dv] [49,50,51].

### 4.3. Duplex Real-Time PCR Assessment

In this study, a duplex real-time PCR system was used to detect the amount of *Pst* during the latent period. After the canopy spectral data were measured, 10 wheat leaves were collected at a contemporary sampling site. Total DNA of wheat leaves was processed as described in [52]. The sequences of the primers and probes refer to reference [53]. The reaction and standard curve of duplex real-time PCR were described in [46]. According to the standard curve, the DNA concentration of *Pst* and wheat were calculated. Then, the molecular disease index (MDI) and the under disease progress curve (AUDPC) could be obtained according to reference [46] and could be used to represent the *Pst* accumulation effect of the development of the disease within a period of time. The higher value of the MDI, the greater the value of AUDPC.

### 4.4. Disease Index Acquisition and Preprocessing

Disease severity was determined by random sampling on the third day after the symptoms appeared. At each sampling point, 10 wheat plants were investigated, and the antepenult leaves and penultimate leaves were sampled for each plant. The survey was conducted every 2 days for a total of three times. Wheat disease investigation concentrated on the disease severity and incidence. Severity (S) refers to the degree of harm caused to plants or plant organs; the grading standard for the severity of WSR refers to reference [54]. The incidence (I) refers to the percentage of diseased plants in the total number of plants and is an indicator of crop disease prevalence. Disease index (DI) reflected the disease severity of wheat population, and it is determined with S and I. The AUDPC was calculated in the same way as above; the larger the value of AUDPC meant that the degree of disease occurrence was also higher.

The correlation between MDI-AUDPC and DI-AUDPC was analyzed using SAS v. 9.0 (SAS Institute INC., Cary, NC, USA) software to ascertain whether the MDI during the latent period could predict DI in the symptomatic period of WSR.

### 4.5. Detection of the MDL Based on the CART Algorithm

Decision tree (DT) is a very flexible classification algorithm. The DT algorithm implements supervised learning; that is, given a sample of a known category, each sample has a set of attributes, and a classifier is obtained through learning, which can apply the correct classification to the new sample. DT is often used to solve classification and regression problems [55]. DT is a hierarchical structure where each node in the tree represents a feature or attribute, and each branch represents a test output. Taking each of the resulting new nodes and repeating the process, the recursion is continued until a stopping criterion is reached, and finally, the leaf node is used as the class label to which the unknown sample belongs. DT is easy to implement, has strong interpretability, and is widely used.

Classification and regression trees (CART) are used to construct a binary decision tree through recursion, which can handle continuous and discrete variables. If the predicted variables are discrete data, CART generates a classification decision tree; if the predicted variables are continuous data, CART generates a regression decision tree. The tree-building process is a process of recursive binary partitioning of the training set, involving the issue of how to select the best division attribute from multiple attributes. CART uses the Gini coefficient to measure the difference in the values of the two sets of attribute variable test outputs. The goal of CART is to reveal the relationships between predictor variables and dependent variables through a recursive portioning algorithm [56]. CART usually presents the final results in the form of graphics, which are easier to understand and analyze than the results produced by other classification methods. Therefore, CART is an intensively used data analysis method in data mining.

Based on 24 testing sets with 6 hyperspectral parameters and 4 modeling ratios and 6 complete datasets with 6 hyperspectral features parameters, the DT analysis was carried out. The CART algorithm in DT was used to detect the MDL of *Pst*. The two categories with the smallest MDI values in the DT analysis were selected, and their intersection point was found according to the normal distribution equation. The analysis was implemented using SAS v. 9.0 (SAS Institute INC., Cary, NC, USA) software.

### 4.6. Classification Model Based on the MDL

On the sampling points, the *Pst* content and the canopy hyperspectral data were obtained at the same time, and the hyperspectral data were matched with MDI point-to-point. The MDI was converted into a classification label of the model. Based on different modeling ratios and different hyperspectral transformation parameters, the classification model of the minimal value of MDI was established, and the accuracy of the model for WSR in latent period based on MDL was verified. The classification models were set up on the WEKA platform using three machine learning algorithms, namely, function-logistic, lazy-IBK, and meta-random committee, and evaluated the classification performance of the three classifiers. The WEKA platform is a data mining system developed by Waikato University in New Zealand. It provides data preprocessing and algorithm performance evaluation methods suitable for various datasets and has strong scalability and compatibility [57].

Linear regression mainly uses predetermined weights to linearly combine independent variables, and the dependent variables are quantitative data. However, for classification problems, the dependent variable was qualitative data, so logistic regression analysis was required; that was, the result of linear regression was mapped to the activation function (sigmoid function), and the result was no longer limited to 0–1 but could be any value between negative infinity and positive infinity so as to avoid generating illegal probability values.

Lazy-IBK is a linear search method in which a supervised learning algorithm is used to determine the class of the output data based on the Euclidean distances of the k nearest neighbors [58]. If K = 1, the new data is assigned to the class of its nearest neighbors. This algorithm is a simple and easy way to implement a classification algorithm.

This algorithm can generate a set of randomized base classifiers. Each classifier uses a different random seed, but they use the same data. Then, the final prediction result is the average of the predictions generated by the set of base classifiers, which reduces the computational errors and statistical errors [59].

### 4.7. Model Evaluation

**Figure 9:**
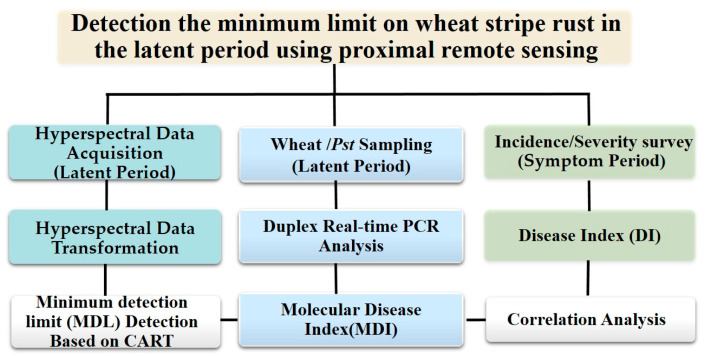
Flowchart.

The credibility evaluation of the results is very important in the process of data mining. This study introduces 10-fold cross-validation, which randomly divides the data into 10 parts. One part of the data was used as the test set, and the remaining nine parts were used as the training set. The average of the 10 test results was taken as the final forecast result when this process was performed 10 times. The advantage of this method was that the evaluation result had little relationship with the data division method, so the generalization ability of model was improved, and it was not easy to overfit [60]. The framework of this study is shown in Figure 9.

## 5. Conclusions

The results of the present study indicated that it was possible to detect the MDL of the MDI of WSR in the latent period using hyperspectral data. Using a DT’s CART method, the MDL of the MDI that the PRS was able to identify during the latent period was 0.7 and 1.2. In the 325–1075 nm waveband, 30 datasets were conducted with logistic, IBK, and random committee, which were used to construct a quantitative classification model based on different hyperspectral characteristics and 4 modeling ratios. When the MDL was 0.7, the accuracy of IBK outperformed the other 2 algorithms. The optimal model employed R_2nd.dv as the spectral feature and 2:1 as the modeling ratio, and the accuracy of the testing set reached 91.67%. It was suggested that the identification and assessment of the severity of WSR based on the PRS approach were viable. Further experiments should be carried out in the field to validate the correctness of the MDL produced in this work.

## Figures and Tables

**Table 1:** Correlation analysis of different wheat cultivars between MDI-AUDPC and DI-AUDPC.

Cultivar	Correlation Coefficient	Significance Level	Regression Equation	R^2^	Root Mean Square Error
Mingxian169	0.89894	<0.0001	y = 27.19 + 2.2528x	0.8081	5.64583
Jing 0045	0.66312	<0.0001	y = 32.487 + 0.8427x	0.4397	3.74929
Nongda 195	0.58177	<0.0007	y = 80.388 + 6.9942x	0.3385	23.04336

**Table 2:** MDLs of 6 hyperspectral features on 4 modeling ratios on the 24 testing sets.

R	Mean 1	Std. Dev 1	Mean 2	Std. Dev 2	Point
1:1	0.242	0.667	1.687	3.113	1.2729
2:1	0.103	0.464	0.498	1.023	0.7261
3:1	0.099	0.358	0.489	1.112	0.7474
4:1	0.131	0.52	0.358	0.779	0.6513
R_1st.dv	Mean 1	Std. Dev 1	Mean 2	Std. Dev 2	Point
1:1	—	—	—	—	—
2:1	0.235	0.721	1.92	2.662	1.2668
3:1	0.192	0.802	1.81	2.554	1.2034
4:1	0.161	0.683	1.868	2.611	1.1718
R_2nd.dv	Mean 1	Std. Dev 1	Mean 2	Std. Dev 2	Point
1:1	—	—	—	—	—
2:1	0.342	0.935	1.918	2.835	1.3837
3:1	0.239	0.84	1.673	2.565	1.2351
4:1	0.223	0.831	1.258	1.954	1.0659
lg(1/R)	Mean 1	Std. Dev 1	Mean 2	Std. Dev 2	Point
1:1	0.242	0.667	1.687	3.113	1.2826
2:1	0.103	0.464	0.498	1.023	0.7337
3:1	0.099	0.358	0.489	1.112	0.7576
4:1	0.131	0.52	0.358	0.779	0.6365
lg(1/R)_1st.dv	Mean 1	Std. Dev 1	Mean 2	Std. Dev 2	Point
1:1	0.376	1.155	2.752	3.404	1.6469
2:1	0.286	0.863	1.943	2.798	1.3283
3:1	0.448	1.313	2.853	3.445	1.7590
4:1	0.555	1.521	3.549	3.825	2.0269
lg(1/R)_2nd.dv	Mean 1	Std. Dev 1	Mean 2	Std. Dev 2	Point
1:1	0.246	0.715	2.266	3.207	1.3776
2:1	0.302	1.028	1.846	2.635	1.3283
3:1	0.272	0.824	1.795	2.768	1.3143
4:1	0.136	0.472	1.191	1.578	0.9070

**Table 3:** MDLs of different hyperspectral features on the six complete datasets.

Hyperspectral Features	Mean 1	Std. Dev 1	Mean 2	Std. Dev 2	Point
R	0.115	0.470	0.400	0.88	0.6939
R_dv1	0.177	0.751	1.775	2.459	1.1718
R_dv2	0.260	0.939	1.571	2.422	1.2193
lg(1/R)	0.115	0.470	0.400	0.880	0.6939
lg(1/R)_dv1	0.359	1.044	1.206	1.901	1.1560
lg(1/R)_dv2	0.309	0.913	1.646	2.553	1.2984

**Table 4:** Comparison of the MDL between six hyperspectral features and the average of four modeling ratios.

Hyperspectral Features	Point 1	Point 2
R	0.6939	0.7083
R_dv1	1.1718	1.2140
R_dv2	1.2193	1.2282
lg(1/R)	0.6939	0.7092
lg(1/R)_dv1	1.1560	1.6903
lg(1/R)_dv2	1.2984	1.2318

Note: Point 1 was the minimum detection limit of the six hyperspectral features in the complete dataset, and Point 2 was the average of the MDL of the six hyperspectral features under four modeling ratios.

**Table 5:** Classification accuracy of the optimal models based on 3 algorithms in the 325–1075 nm waveband for MDL of 0.7.

Algorithms	Hyperspectral Features	Modeling Ratios (Training:Testing)	Accuracy (%)		
			Training	Testing	Cross Validation
Logistic	lg(1/R)	2:1	100	85.25	86.97
IBK	R_2nd.dv	2:1	100	91.67	90.60
Randomcommittee	lg(1/R)_1st.dv	3:1	100	85.47	88.25

**Table 6:** Classification accuracy of the optimal models based on 3 algorithms in the 325–1075 nm waveband for MDL of 1.2.

Algorithms	Hyperspectral Features	Modeling Ratios (Training:Testing)	Accuracy (%)		
			Training	Testing	Cross Validation
Logistic	R	1:1	100	85.47	80.98
IBK	R_1st.dv	2:1	100	91.03	89.53
Randomcommittee	lg(1/R)_1st.dv	2:1	100	85.90	85.47

## Data Availability

Data are available from the authors upon reasonable request.

## Supplementary Materials

The following supporting information can be downloaded at: https://www.mdpi.com/article/10.3390/plants12152814/s1, Figures S1–S6: Minimum detection limit (MDL) of 6 hyperspectral features on 4 modeling ratios on the 24 testing sets; Figure S7: MDLs of different hyperspectral features on the six complete datasets.
