# Supplementary material for: Detecting the Minimum Limit on Wheat Stripe Rust in the Latent Period Using Proximal Remote Sensing Coupled with Duplex Real-Time PCR and Machine Learning
Source: Plants (Basel). 2023 Jul 29;12(15):2814. doi: 10.3390/plants12152814 (PMC10420842; doi:10.3390/plants12152814)
Supplement: Supplementary file 1 [file plants-12-02814-s001.zip › plants-2478758-supplementary.pdf]

Minimum detection limit (MDL) of six hyperspectral features on four modeling ratios on the 24 testing sets.

1. Analysis of the MDLs for different modeling ratios under the original spectral value (R).

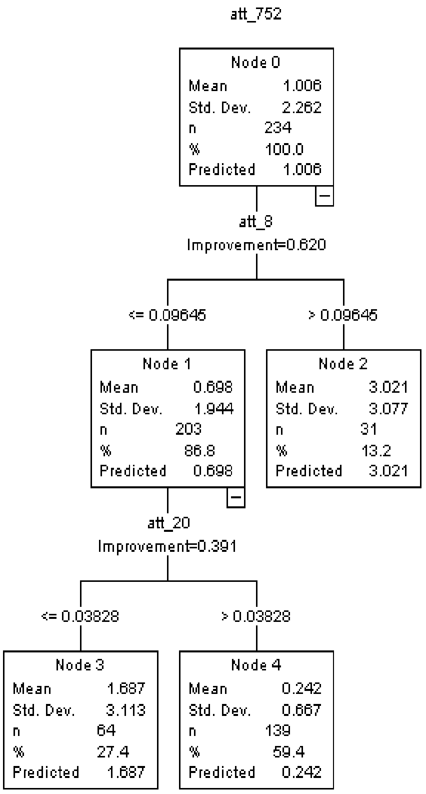

Fig 1-1 The decision tree analysis in modeling 1:1

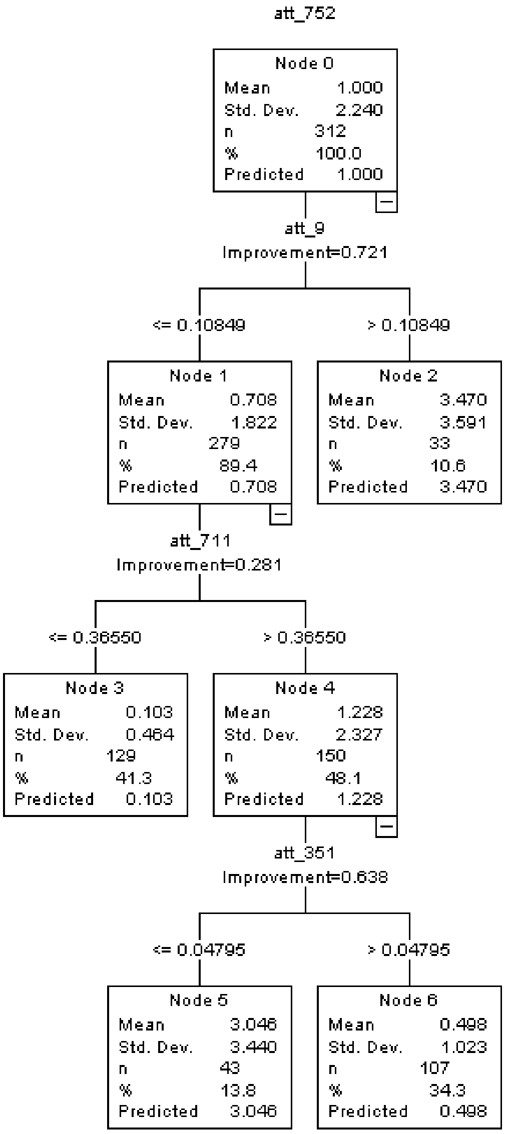

Fig 1-2 The decision tree analysis in modeling 2:1

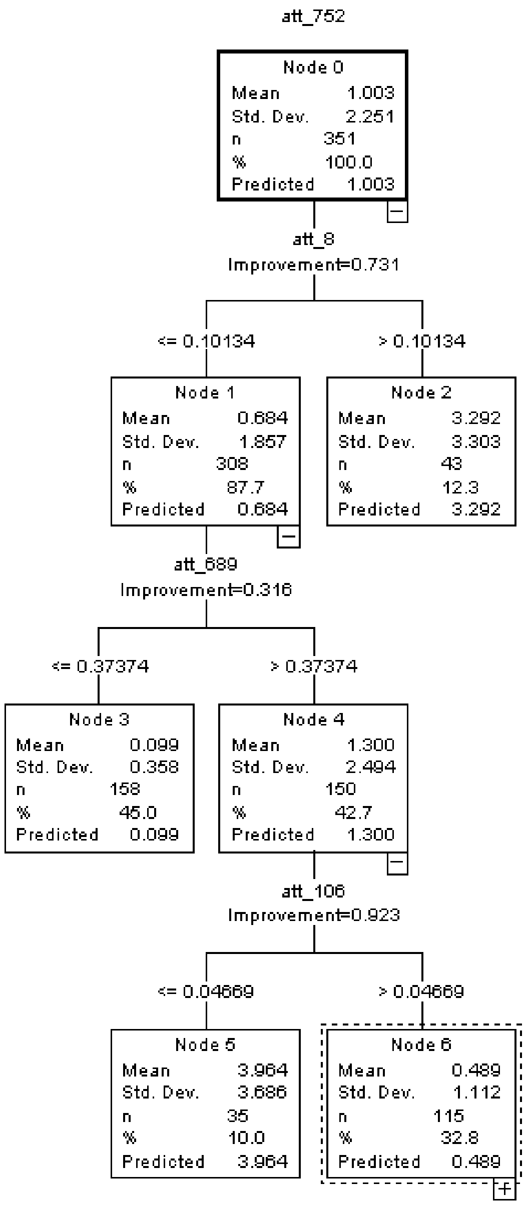

Fig 1-3 The decision tree analysis in modeling 3:1

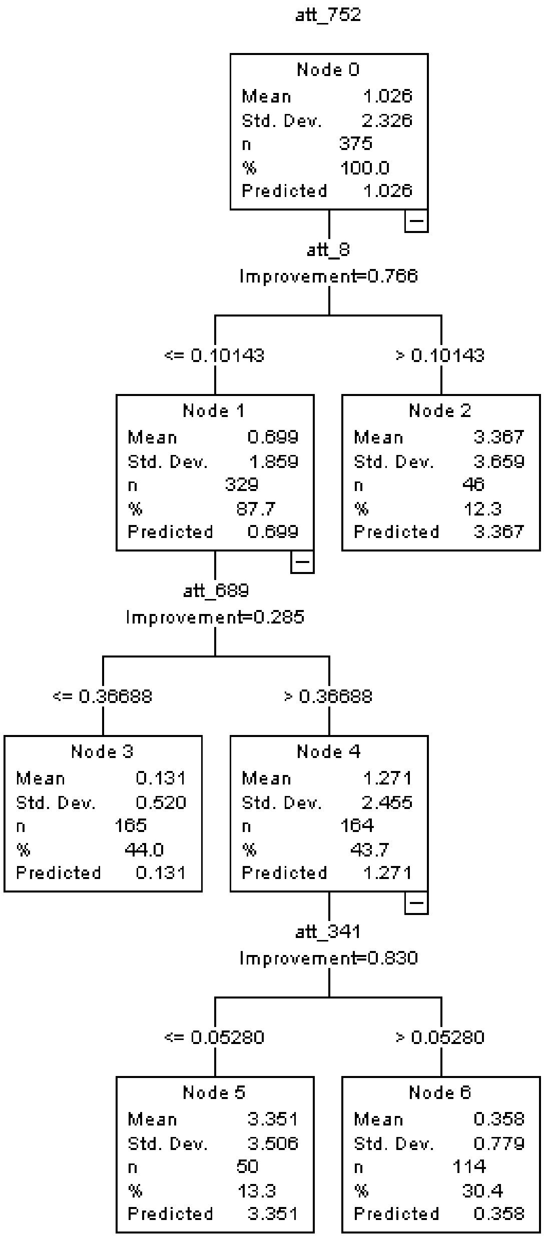

Fig 1-4 The decision tree analysis in modeling 4:1

2. Analysis of the MDL for different modeling ratios under the R_1st.dv.

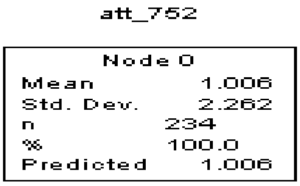

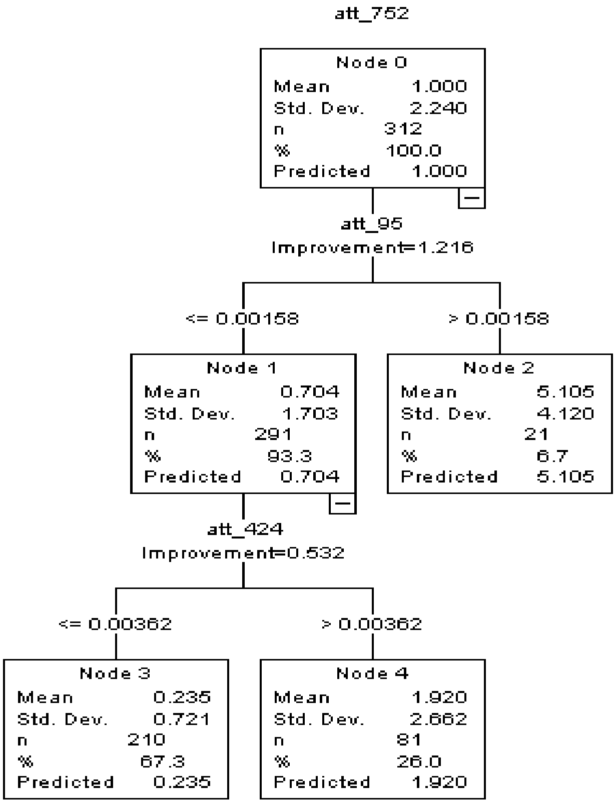

Fig 2-1 The decision tree analysis in modeling 1:1

Fig 2-2 The decision tree analysis in modeling 2:1

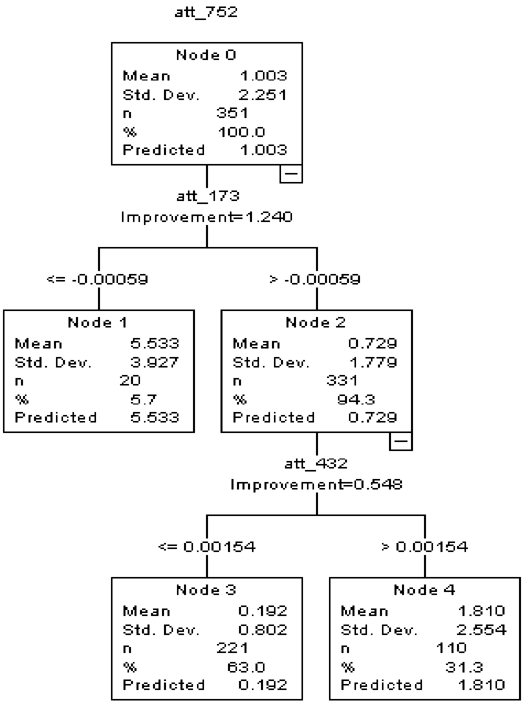

Fig 2-3 The decision tree analysis in modeling 3:1

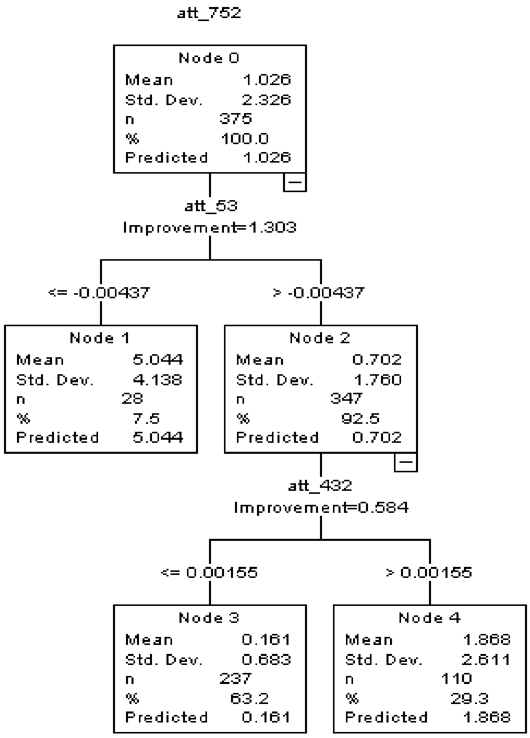

Fig 2-4 The decision tree analysis in modeling 4:1

3. Analysis of the MDL for different modeling ratios under the R_2nd.dv.

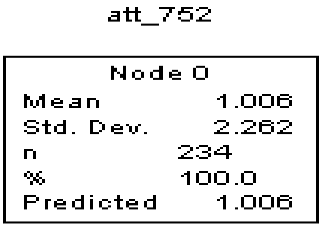

Fig 3-1 The decision tree analysis in modeling 1:1

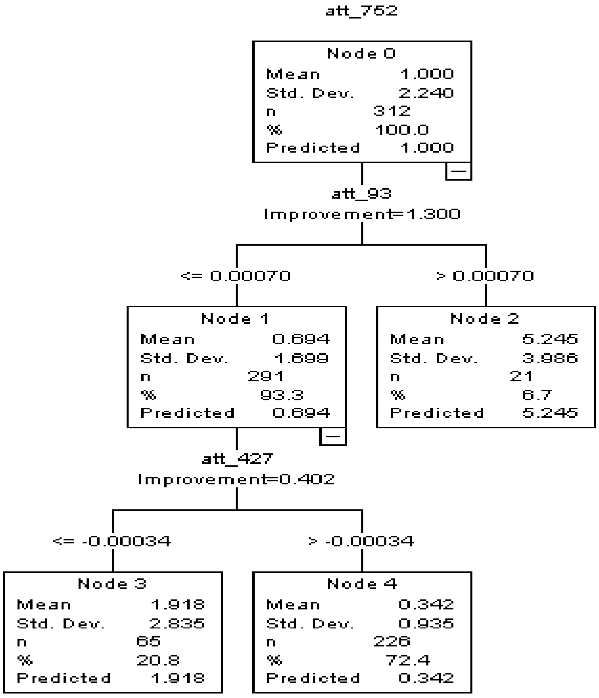

Fig 3-2 The decision tree analysis in modeling 2:1

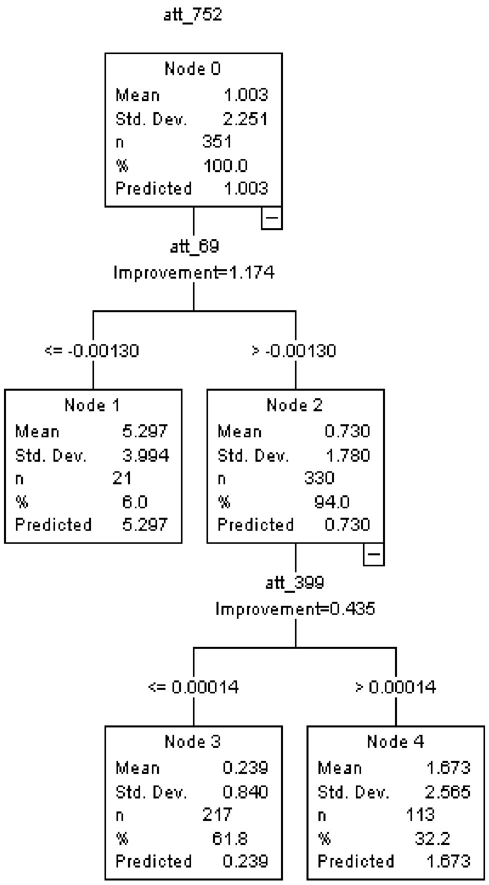

Fig 3-3 The decision tree analysis in modeling 3:1

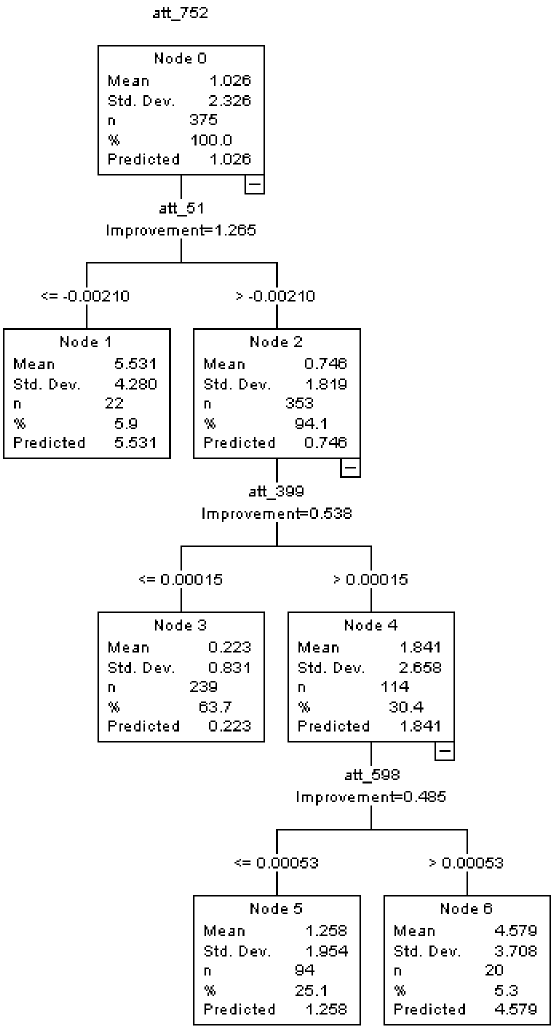

Fig 3-4 The decision tree analysis in modeling 4:1

4. Analysis of the MDL for different modeling ratios under the lg(1/R).

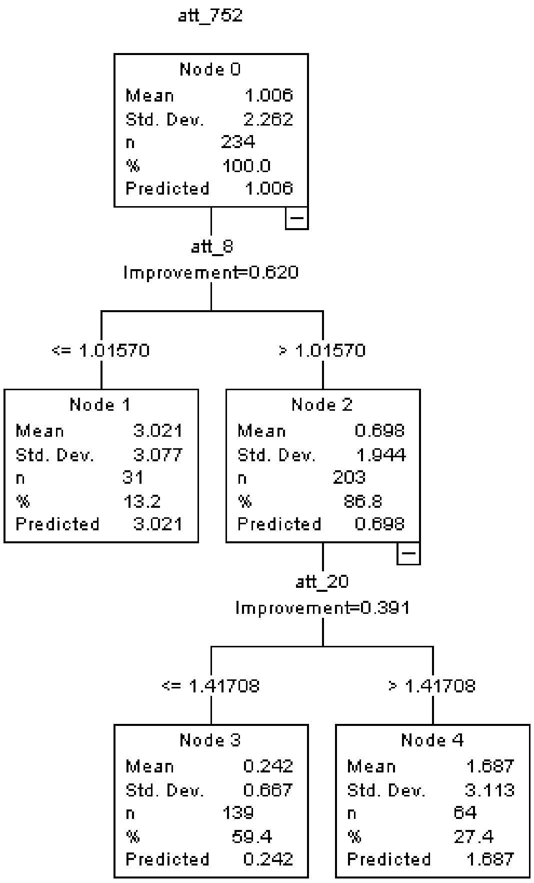

Fig 4-1 The decision tree analysis in modeling 1:1

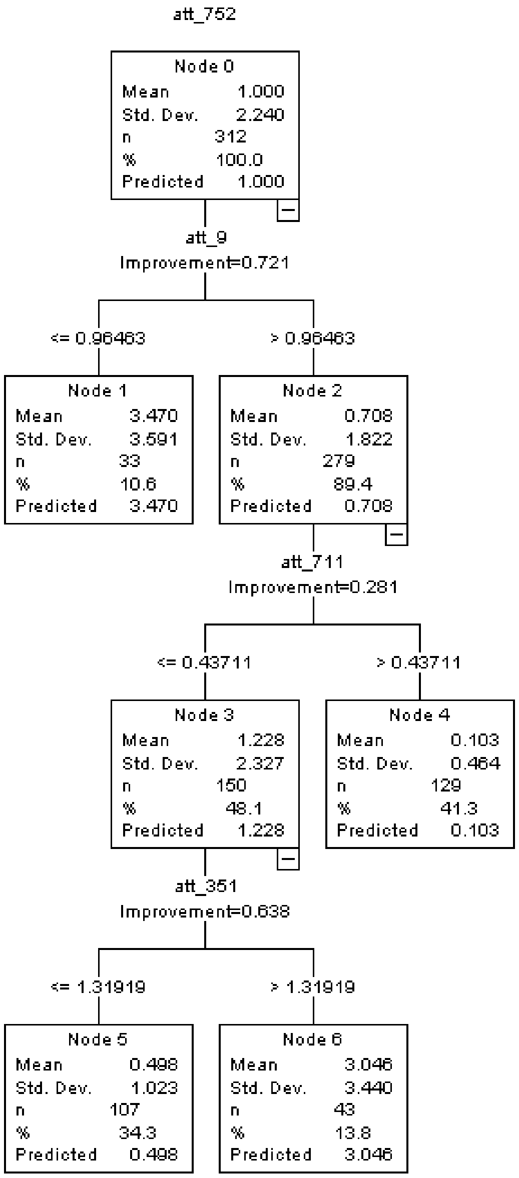

Fig 4-2 The decision tree analysis in modeling 2:1

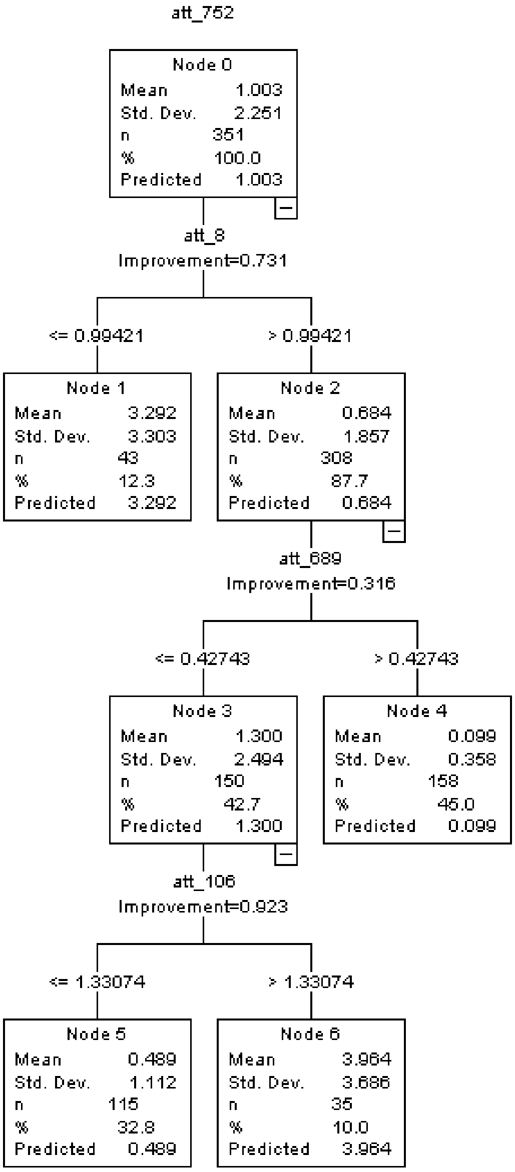

Fig 4-3 The decision tree analysis in modeling 3:1

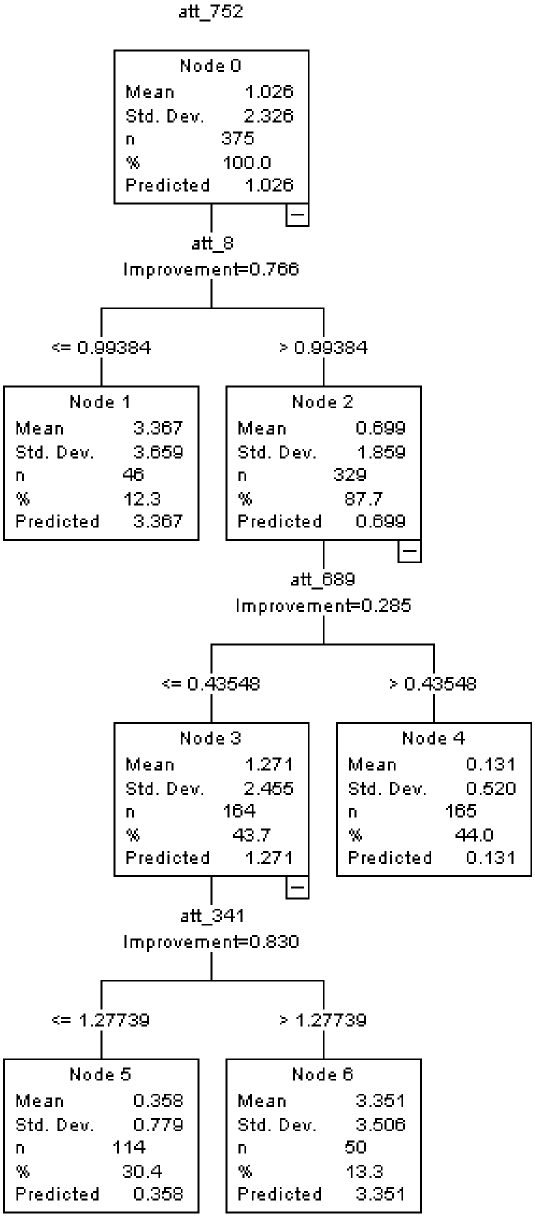

Fig 4-4 The decision tree analysis in modeling 4:1

5. Analysis of the MDL for different modeling ratios under the lg(1/R)_1st.dv]:

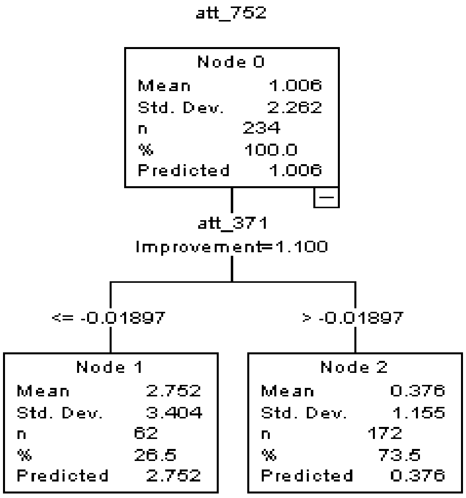

Fig 5-1 The decision tree analysis in modeling 1:1

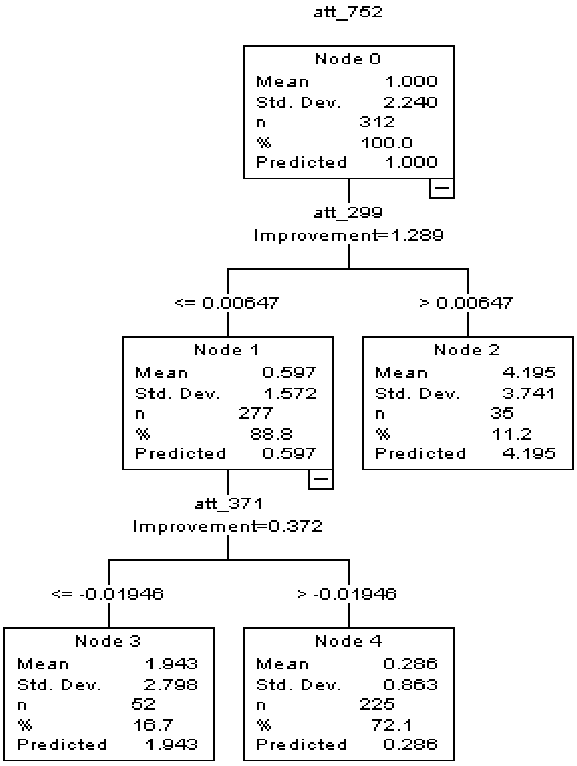

Fig 5-2 The decision tree analysis in modeling 2:1

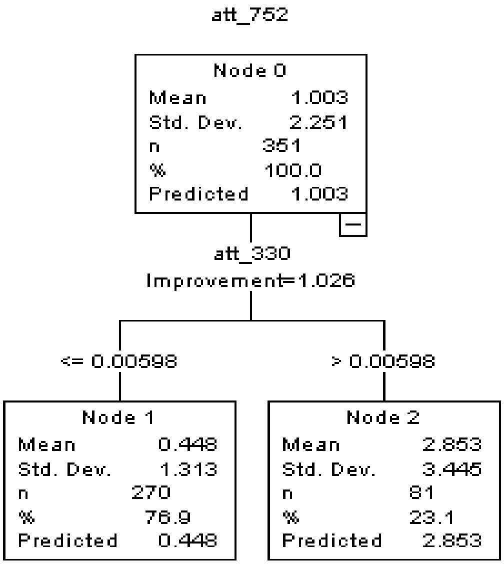

Fig 5-3 The decision tree analysis in modeling 3:1

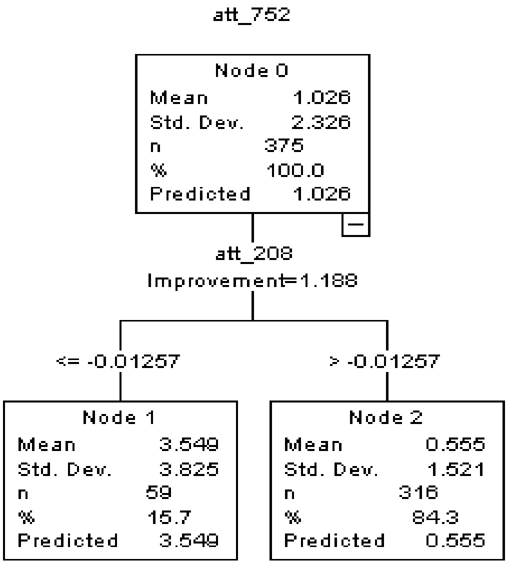

Fig 5-4 The decision tree analysis in modeling 4:1

6. Analysis of the MDL for different modeling ratios under the lg(1/R)_2nd.dv：

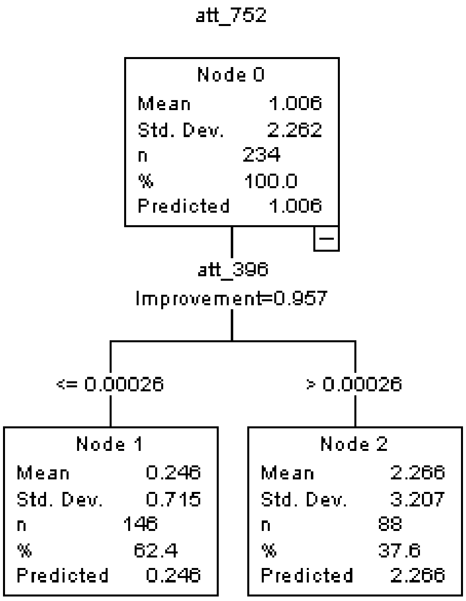

Fig 6-1 The decision tree analysis in modeling 1:1

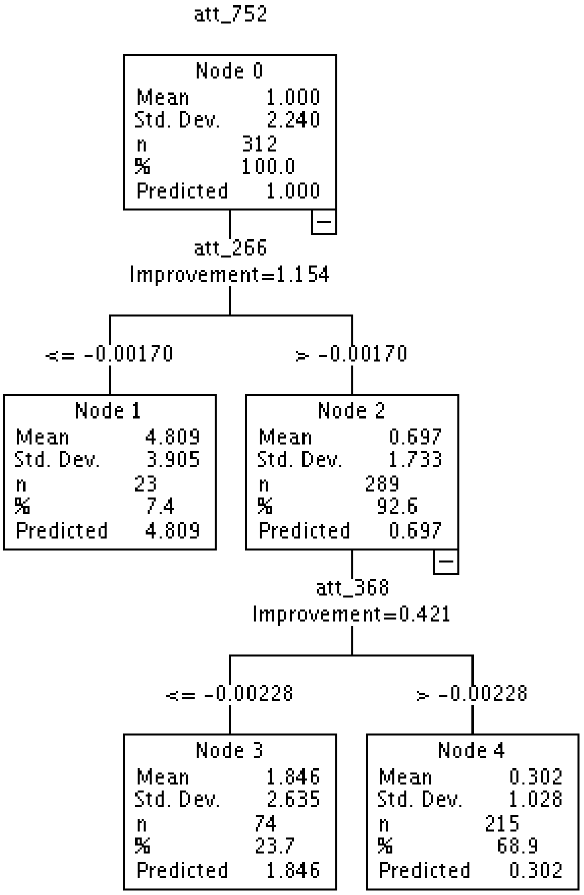

Fig 6-2 The decision tree analysis in modeling 2:1

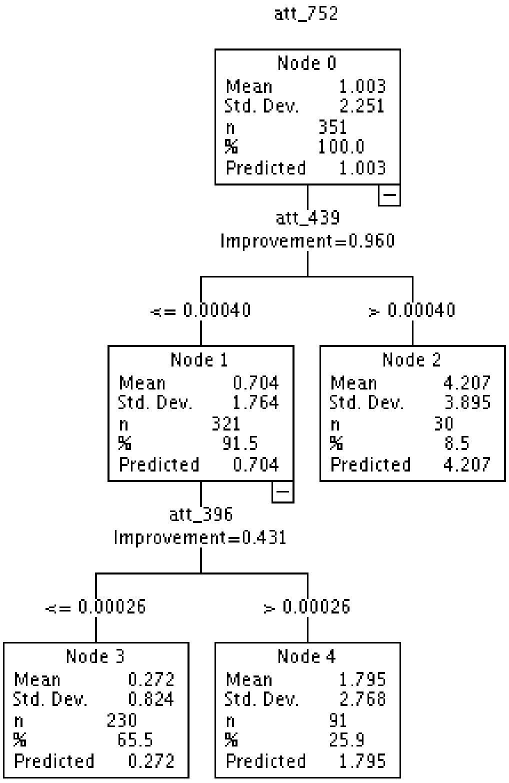

Fig 6-3 The decision tree analysis in modeling 3:1

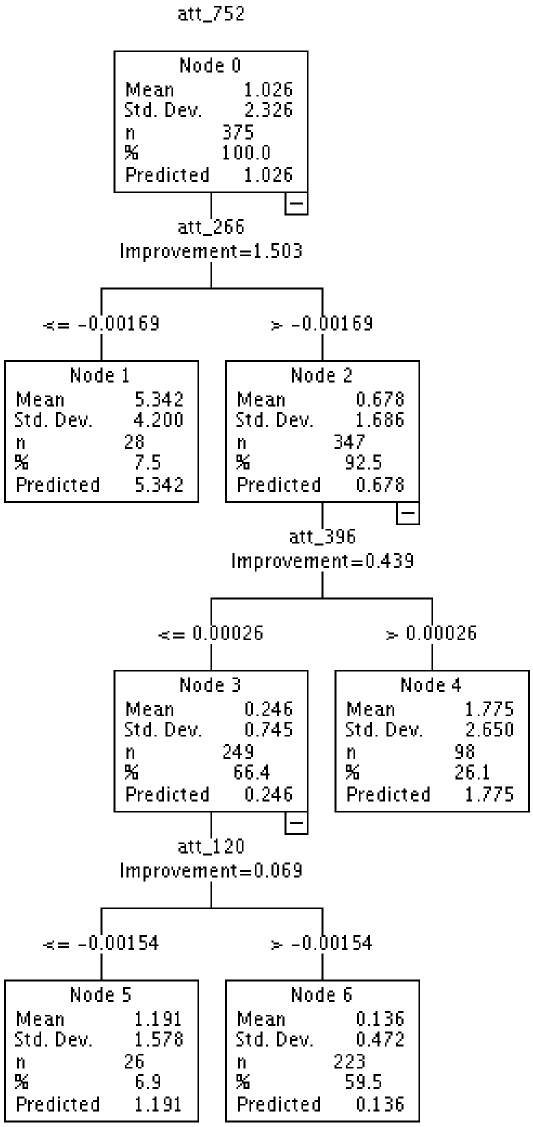

Fig 6-4 The decision tree analysis in modeling 4:1

MDLs of different hyperspectral features on the six complete datasets:

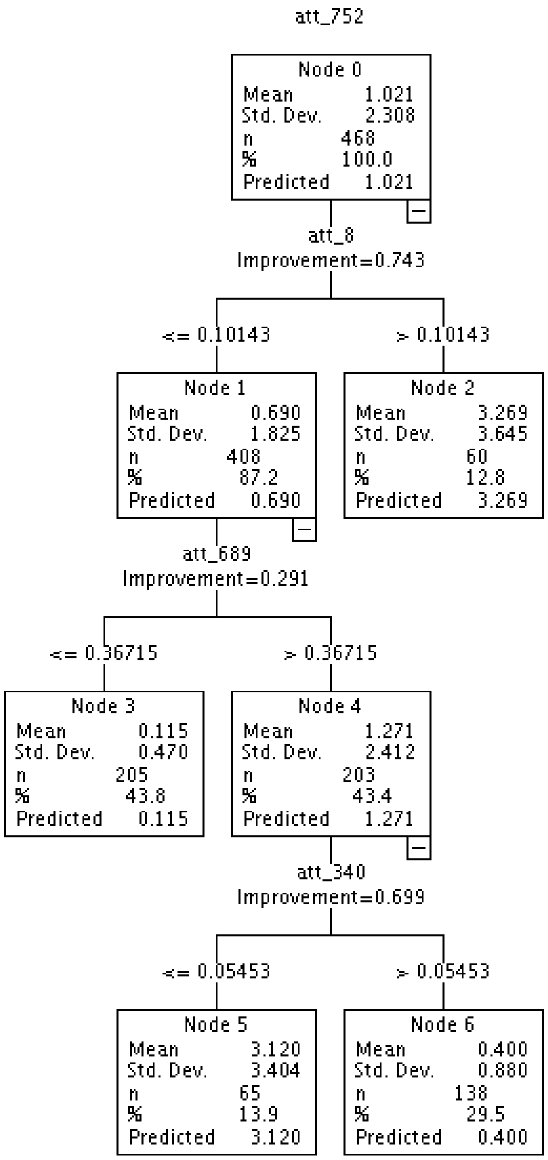

Fig 7-1 The decision tree analysis of R

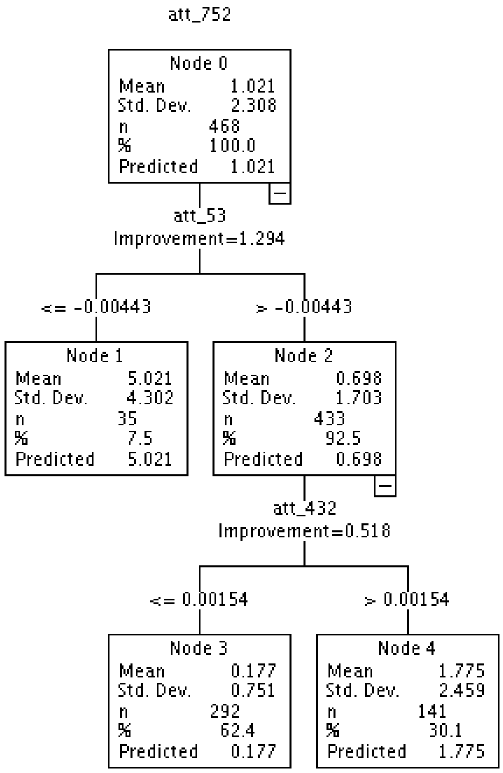

Fig 7-2 The decision tree analysis of R-dv1

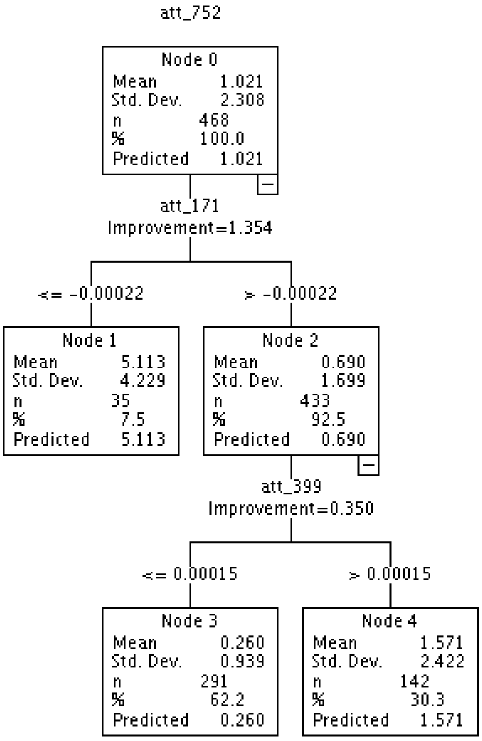

Fig 7-3 The decision tree analysis of R-dv2

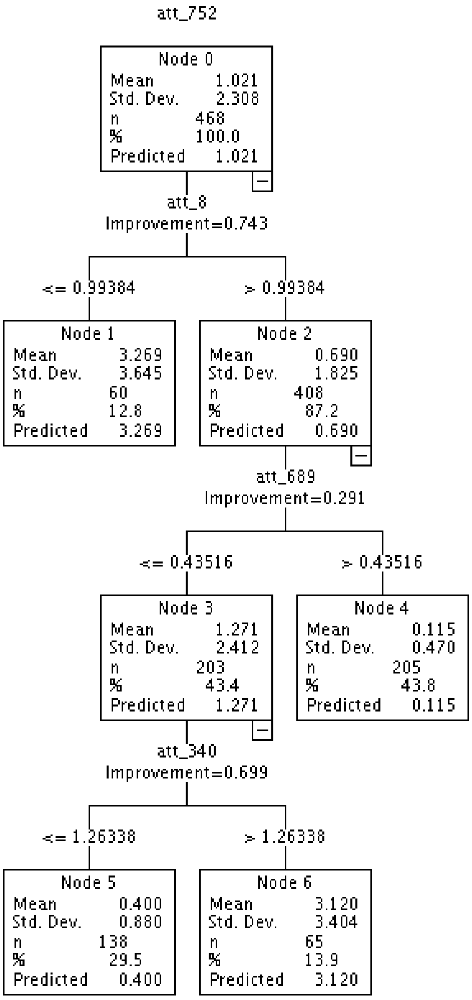

Fig 7-4 The decision tree analysis of R-abs

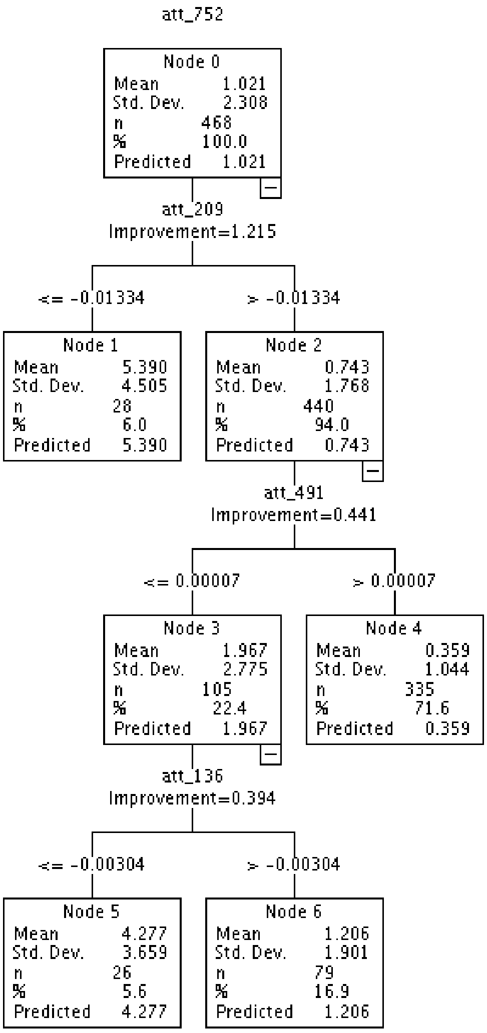

Fig 7-5 The decision tree analysis of R-abs-dv1

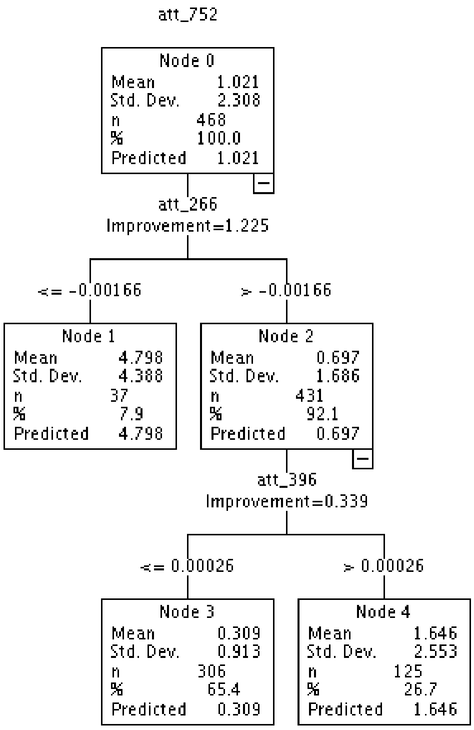

Fig 7-6The decision tree analysis of R-abs-dv2
